# Peritoneal Inclusion Cyst Mimicking Adrenal Pathology in a Young Man: A Rare Case Report

**DOI:** 10.1155/crie/9955929

**Published:** 2025-12-17

**Authors:** Faezeh Sehatpour, Nekoo Panahi, Farid Kosari, Shirzad Nasiri, Maryam Panahi, Amir Reza Radmard, Mahnaz Pejman Sani

**Affiliations:** ^1^ Endocrinology and Metabolism Research Center, Endocrinology and Metabolism Clinical Sciences Institute, Tehran University of Medical Sciences, Tehran, Iran, tums.ac.ir; ^2^ Department of Endocrinology, Shariati Hospital, School of Medicine, Tehran University of Medical Sciences, Tehran, Iran, tums.ac.ir; ^3^ Metabolic Disorders Research Center, Endocrinology and Metabolism Molecular-Cellular Sciences Institute, Tehran University of Medical Sciences, Tehran, Iran, tums.ac.ir; ^4^ Department of Pathology, Shariati Hospital, School of Medicine, Tehran University of Medical Sciences, Tehran, Iran, tums.ac.ir; ^5^ Department of Surgery, Shariati Hospital, School of Medicine, Tehran University of Medical Sciences, Tehran, Iran, tums.ac.ir; ^6^ Department of Pediatric Surgery, Tehran University of Medical Sciences, Tehran, Iran, tums.ac.ir; ^7^ Department of Radiology, Shariati hospital, Tehran University of Medical Sciences, Tehran, Iran, tums.ac.ir

**Keywords:** adrenal cyst mimic, case report, histopathology, peritoneal inclusion cyst, retroperitoneal cyst

## Abstract

Peritoneal inclusion cysts (PICs) are rare, benign, and fluid‐filled lesions most commonly seen in women of reproductive age, typically associated with a history of abdominal surgery, trauma, or inflammatory conditions. Occurrence in males, especially without predisposing factors, is exceedingly uncommon and can pose significant diagnostic challenges due to their nonspecific clinical and radiological features. We report the case of a 19‐year‐old male who presented with chronic right lower quadrant abdominal pain persisting for 4 months. Initial sonography revealed an 80 × 50 mm cystic lesion between the liver and kidney, with subsequent imaging—including contrast‐enhanced computed tomography and magnetic resonance imaging—suggesting an adrenal cyst. Functional and serological workups for adrenal and hydatid pathology were negative. Due to the lesion’s size and persistent symptoms, surgical excision was performed. Intraoperatively, the cyst was found in the retroperitoneal space adjacent to, but not adherent to, the adrenal gland. Histopathological and immunohistochemical analyses confirmed the diagnosis of a PIC. The patient’s postoperative recovery was uneventful. This case illustrates the diagnostic complexity of PICs in atypical patient populations. The lesion’s radiological resemblance to adrenal or other retroperitoneal cystic masses led to initial misdiagnosis and extensive workup. Literature review reveals very few similar cases in males without prior surgery or inflammation. PICs should be considered in the differential diagnosis of cystic abdominal lesions, even in young males without typical risk factors. Accurate diagnosis relies on a combination of clinical assessment, imaging, and histopathological evaluation to ensure appropriate management.

## 1. Introduction

Peritoneal inclusion cysts (PICs) are uncommon, benign, and fluid‐filled lesions that predominantly affect women of reproductive age. They account for ~3%–5% of all peritoneal mesothelial lesions [1].

The normal peritoneum plays a vital role in the transport and absorption of abdominal fluid. However, when its integrity is compromised due to injury or inflammation, its absorptive capacity diminishes. This disruption can initiate a reactive process characterized by proliferation of peritoneal tissue in response to intra‐abdominal inflammation or trauma, ultimately resulting in the formation of cystic structures [2].

They often develop following abdominal or pelvic surgery, trauma, inflammatory bowel disease, radiotherapy, infection, or conditions such as endometriosis. Although most cases occur in adult women, a small number have been reported in adolescents and young patients. Clinically, PICs typically present with nonspecific symptoms, including abdominal pain, pelvic pressure, or palpable masses. However, ~10% of cases are incidentally discovered during imaging studies or surgical procedures.

The differential diagnosis includes paraovarian cysts, hydrosalpinx, and low‐grade cystic mesothelioma. These lesions often exhibit nonspecific clinical presentations and imaging characteristics that can closely mimic other abdominal pathologies, making accurate diagnosis challenging. Consequently, further evaluation is essential to distinguish PICs from other cystic structures within the abdomen or pelvis [3–5]. Although PICs are benign, they carry a risk of morbidity due to potential infection and recurrence, often necessitating repeated surgical interventions. Management typically involves subcutaneous drainage or surgical removal, which can be performed via laparoscopy or laparotomy to achieve complete cyst excision [5–8].

We report the case of a young man who presented with chronic abdominal pain and initial imaging findings suggestive of an adrenal cyst; however, histopathological examination ultimately confirmed the diagnosis of a large PIC.

## 2. Case Presentation

A 19‐year‐old male presented to our hospital with a chief complaint of right lower quadrant abdominal pain persisting for 4 months. The pain, which began during military training, was severe and intermittent. He had consulted multiple physicians, and during a diagnostic workup at another medical center, an ultrasound revealed an 82 × 57 mm lesion in segment six of the liver. Based on these findings, he was referred to a hepatobiliary surgeon for further evaluation. His past medical history was unremarkable, with no known comorbidities or prior abdominal surgeries. The patient denied any history of unprotected sex or genital infections.

On initial physical examination in the emergency department, the patient’s vital signs were as follows: blood pressure 90/65 mmHg, pulse rate 98 beats per minute, temperature 37.5°C, and oxygen saturation 98% on room air. Abdominal examination revealed mild tenderness localized to the right lower quadrant without rebound tenderness. Rectal examination detected fecal material. No genital lesions or other signs suggestive of sexually transmitted infection were noted on examination. Examination of other systems was also unremarkable. Primary laboratory data are summarized in Table 1.

**Table 1:** Primary laboratory data

Test	Reference range	Patient result	Test	Reference range	Patient result
WBC ( × 1000/mm³)	4500–12,500	7500	AST (U/L)	< 37	19
HB (g/dL)	14–18	14.3	ALT (U/L)	< 40	7
MCV (fL)	45–52	84.91	ALK. P (U/L)	80–300	251
PLT ( × 1000/mm³)	80–100	362	T. Bili (mg/dL)	< 1.1	0.5
BUN (mg/dL)	8–20	8	D. Bili (mg/dL)	< 0.2	0.2
Cr (mg/dL)	0.7–1.4	0.7	Lipase (U/L)	< 60	21

Abbreviations: ALK.P, alkalinephosphatase; ALT, alanine aminotransferase; AST, aspartate aminotransferase; BS, blood sugar; BUN, blood urea nitrogen; Cr, creatinine; D.Bili, direct bilirubin; ESR, erythrocyte sedimentation rate; HB, hemoglobin; K, potassium; LDH, lactate dehydrogenase; MCV, mean corpuscular volume; Na, sodium; PLT, platelet; T.Bili, total bilirubin; WBC, white blood cell.

**Figure 1:**
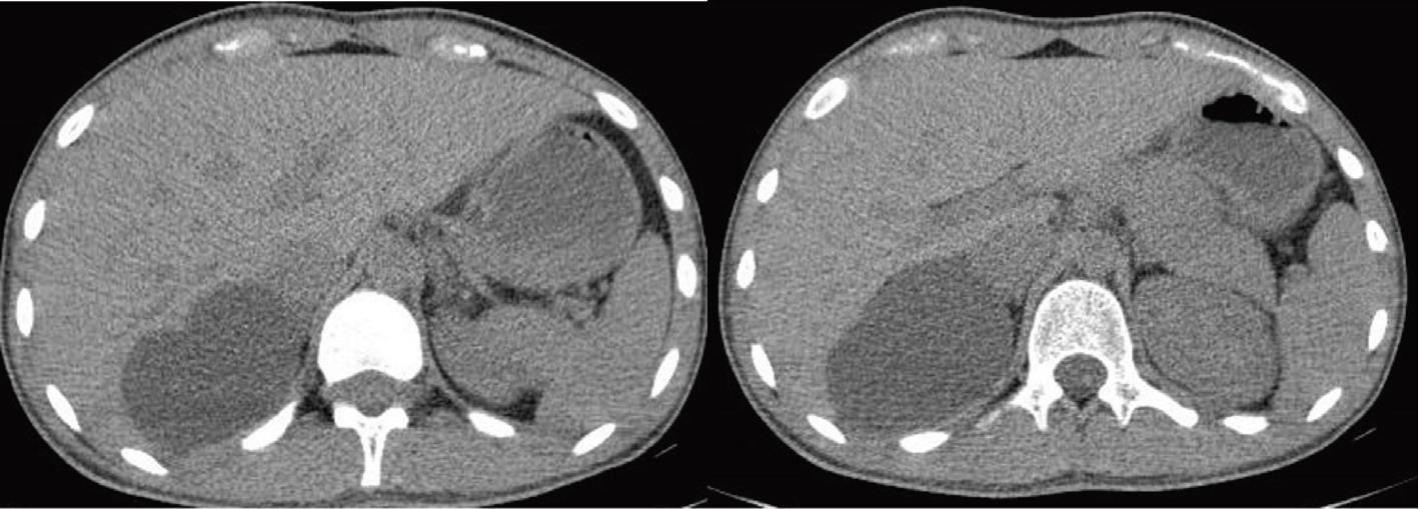
Sagittal CT image showing an 80 × 50 mm cystic lesion located primarily between the liver and right kidney. The lesion causes compression of the right liver lobe.

Due to the patient’s persistent abdominal pain, sonography was initially performed, revealing an 80 × 50 mm cystic structure with posterior acoustic enhancement located between the liver and kidney, suggestive of a hepatic origin. To further characterize the lesion, contrast‐enhanced computed tomography (CT) was conducted, confirming an 80 × 50 mm well‐defined cystic lesion in the same region and raising the possibility of a hydatid cyst among the differential diagnoses (Figure 1). Given the overlapping imaging features of cystic lesions in this area, further evaluation with magnetic resonance imaging (MRI) was pursued. MRI suggested that the cyst originated from the adrenal gland rather than the liver (Figure 2). Functional imaging with metaiodobenzylguanidine (MIBG) showed no avid lesion. Additionally, serology for hydatid cyst and blood and urine cultures were negative.

Figure 2Large right retroperitoneal unilocular cystic lesion (83 × 72 × 60 mm) arising from the right adrenal gland, shown on MRI. axial T1 (A), T2 (B), weighted sequences and coronal (C) views are presented. The lesion exhibits a smooth wall and homogeneous signal without internal enhancement, septations, solid components, or calcifications.

**Figure:**
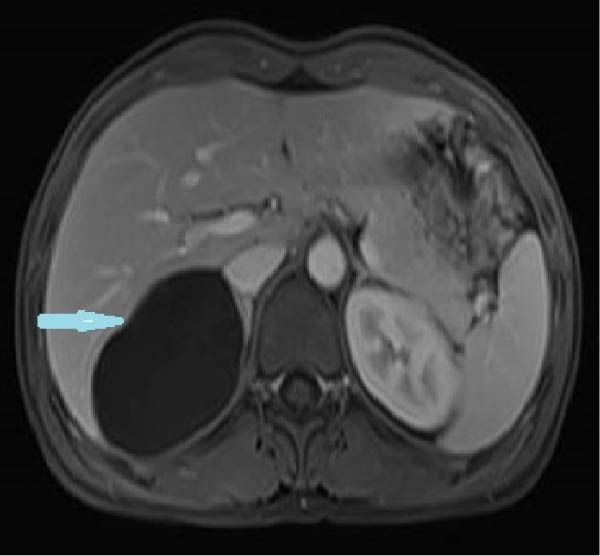
(A)

**Figure:**
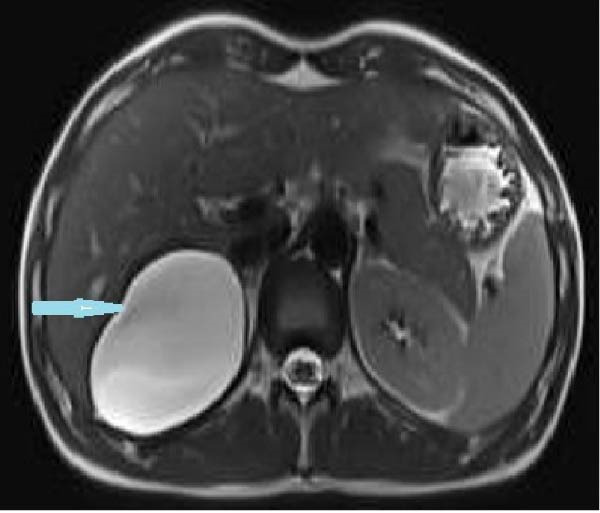
(B)

**Figure:**
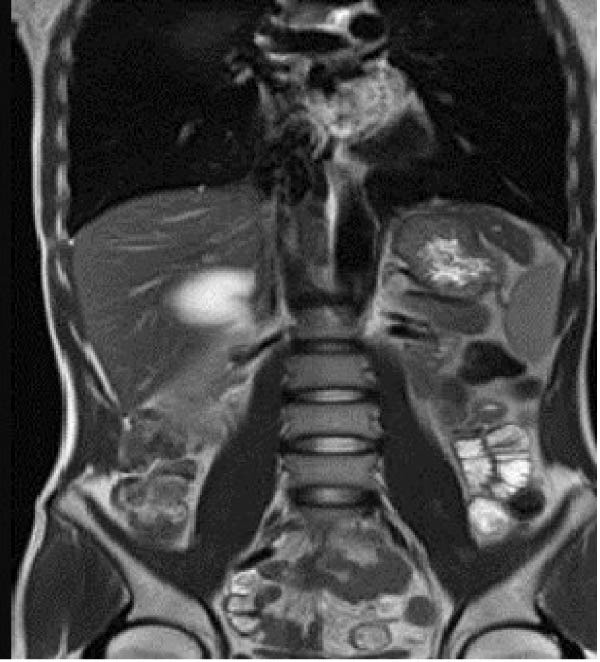
(C)

Due to suspicion of an adrenal origin of the lesion, a comprehensive functional hormonal assay was performed to assess its secretory activity (Table 2).

**Table 2:** Functional hormonal assay results

Hormone/Test plasma	Reference range	Patient result	Hormone/Test 24 h urine	Reference range	Patient result
Cortisol (µg/dL) (ODS)	< 1.8	< 0.8	Metanephrine (µg)	< 350	307
DHEA‐S (ng/mL)	45.1–385	300.5	Normetanephrine (µg)	< 900	289
17‐Hydroxyprogesterone (ng/mL)	0.2–3.1	0.23	Epinephrine (µg)	< 20	15
Aldosterone (ng/dL)	5.3–99.1	51.3	Norepinephrine (µg)	< 90	30
Renin (ng/dL)	3.7–43.2	10.4	VMA (mg)	< 13.6	2.5

Abbreviations: DHEA‐S, dehydroepiandrosteronesulfate; HRS, hours; ODS, overnight dexamethasone test; VMA, vanillylmandelic acid.

Given the tumor’s size and the patient’s symptomatic presentation, surgical removal was deemed the most appropriate treatment. Preoperative preparation included administration of the alpha‐blocker phenoxybenzamine due to suspicion of a nonsecretory cystic pheochromocytoma. The dosage was carefully titrated based on the patient’s blood pressure and the presence of orthostatic hypotension.

**Figure 3:**
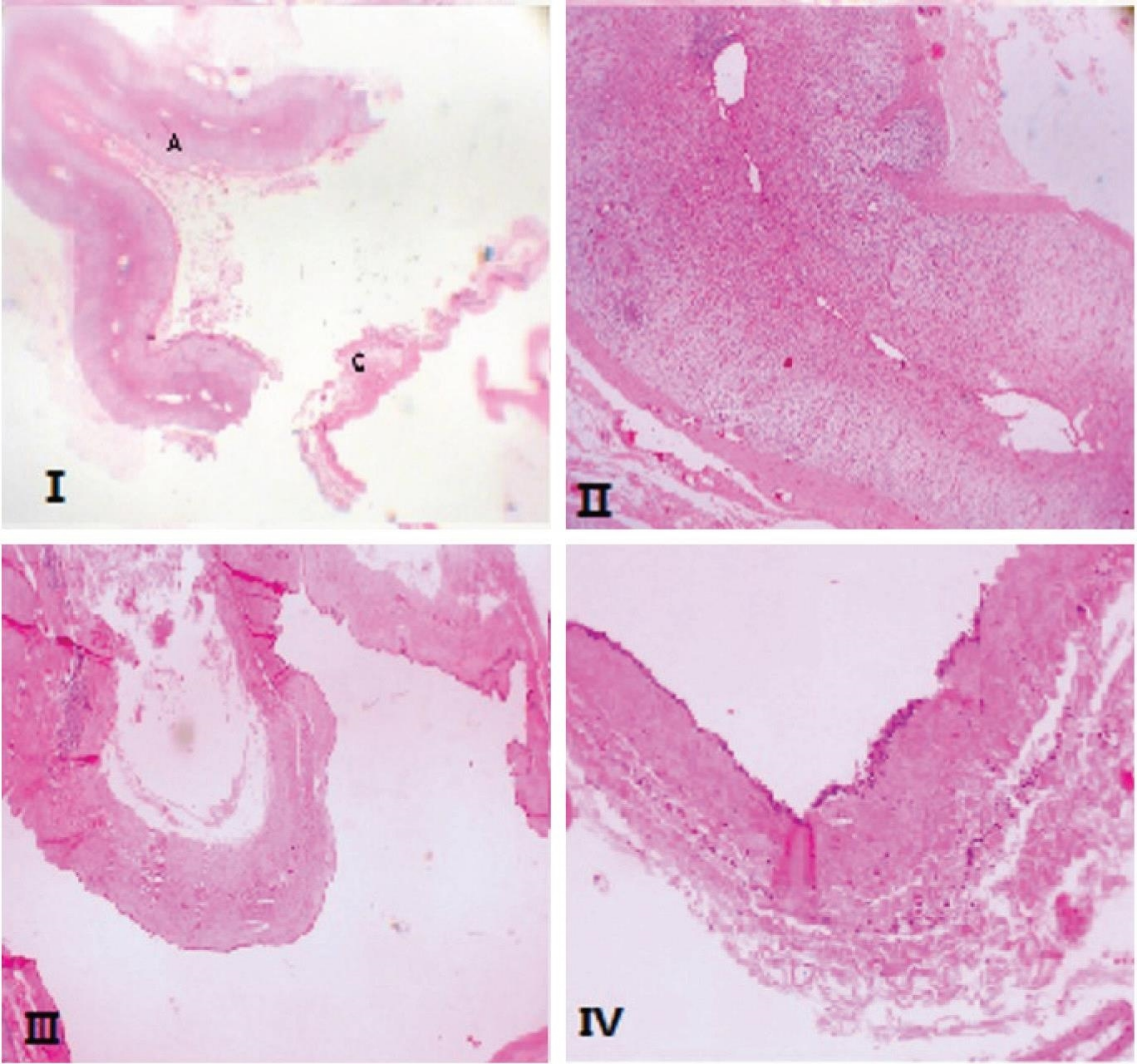
Ⅰ, panoramic view of the resected adrenal gland (A) and separated cyst (C); Ⅱ, Low power view of normal adrenal gland (H&E × 40); Ⅲ, Low power view of the cyst with epithelial lining (H&E × 40); Ⅳ, High power view of the cyst lining showing cuboidal lining and underlying dense fibroconnective tissue (H&E × 200).

**Figure 4:**
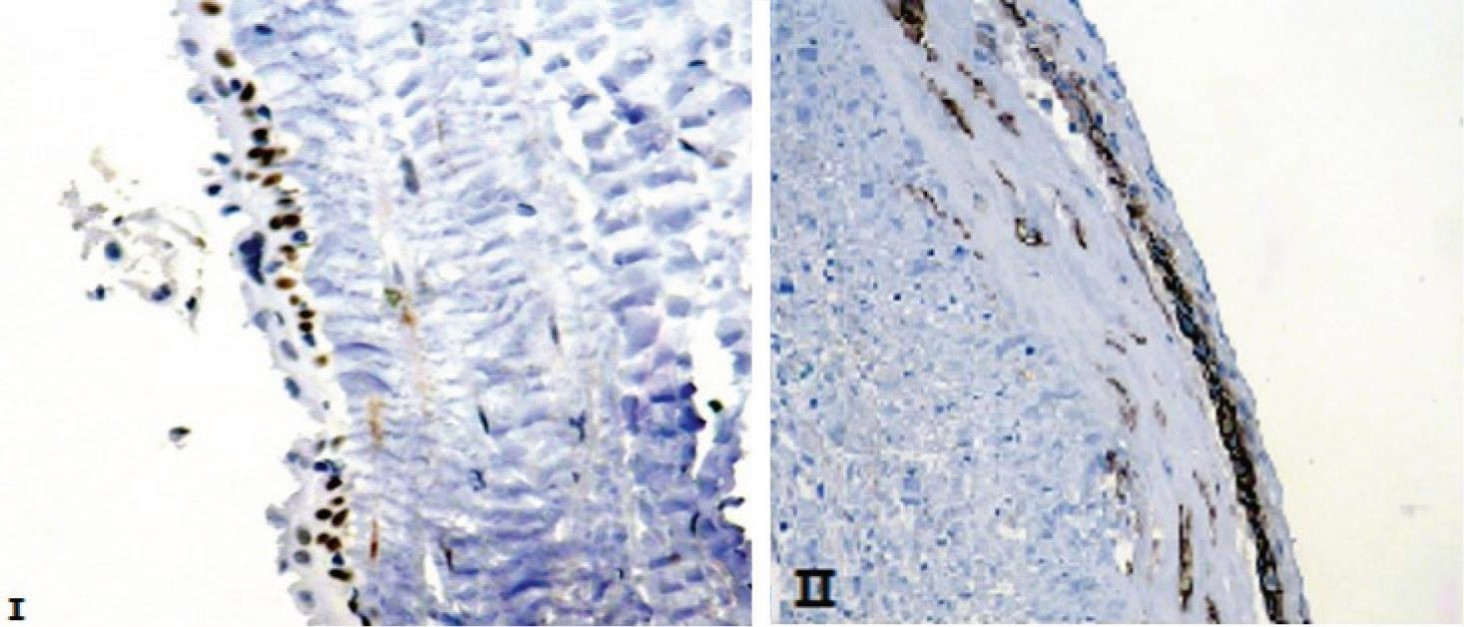
Ⅰ, Positive immunohistochemical staining of the overlying mesothelial cells for WT1 ( × 400); Ⅱ; Positive immunohistochemical staining of the overlying mesothelial cells for D2−40 ( × 200).

On November 27, 2024, the patient underwent surgical resection of the cyst along with the right adrenal gland. Intraoperatively, the lesion was identified in the retroperitoneal space adjacent to, but not adherent to, the adrenal gland. Macroscopic evaluation confirmed that the cyst wall was separate from the adrenal tissue (Figure 3). Immunohistochemical analysis showed positivity for WT1 and D2−40, supporting the diagnosis of a PIC (Figure 4). The patient’s postoperative course was uneventful, and he was discharged in stable condition. At the follow‐up visit, the patient remained asymptomatic, with no evidence of disease recurrence.

## 3. Discussion

PICs are rare, benign lesions that pose diagnostic challenges due to their resemblance to other cystic abdominal or pelvic masses. Clinically, they may present with a wide spectrum of symptoms—from nonspecific abdominal or pelvic pain to being entirely asymptomatic. The size of these lesions can vary significantly, ranging from small, localized fluid collections to large cystic structures occupying the pelvis and lower abdomen [5, 9].

PICs predominantly affect females, especially those in their third or fourth decades of life, and are frequently associated with a history of prior abdominal surgery, trauma, or underlying inflammatory conditions. In contrast, our case involves a young male patient with no history of abdominal surgery, trauma, or inflammatory disease, representing an uncommon presentation. Such cases in males are exceedingly rare, with only a few reported in the literature [1, 5, 10, 11] .

For instance, Killoran et al. described a 26‐year‐old man presenting with chronic abdominal pain and no significant past medical history, initially misdiagnosed with pseudomyxoma peritonei. Diagnostic laparoscopy revealed mesothelial lining cells positive for calretinin and WT1 on IHC staining. Subsequent surgery identified multiple cysts at the appendix tip and pelvic cavity, with IHC confirming PICs [1].

Similarly, a 2022 case report detailed a 16‐year‐old male without notable medical history who presented with lower abdominal pain and urinary symptoms. Imaging revealed multiple thin‐walled cystic lesions, up to 7.5 cm, located between the bladder and rectum. Laparoscopic excision showed free‐floating, non‐adherent cysts, and histopathology confirmed multilocular PICs [12].

PICs can easily be mistaken for other pathologies, such as hydatid cysts, retroperitoneal tumors, or cystic lesions of adrenal or solid organs, due to overlapping imaging features and anatomical proximity [13–15]. In our case, initial radiological assessments suggested an adrenal cyst, likely because of the lesion’s location near the adrenal gland and its imaging characteristics, which closely mimicked adrenal pathology. This led to further evaluation and workup for an adrenal‐origin lesion, highlighting the diagnostic challenge PICs can pose. These diagnostic pitfalls underscore the importance of including PICs in the differential diagnosis when evaluating cystic lesions in the abdomen or pelvis, especially when imaging findings are atypical or inconclusive.

Management of PICs is guided by symptom severity, cyst size, and the risk of recurrence. Asymptomatic patients are typically managed conservatively with serial imaging and observation. Minimally invasive options, such as image‐guided aspiration or sclerotherapy, may provide temporary relief but are associated with a high risk of recurrence. For large cysts, symptomatic cases, or when malignancy cannot be excluded, complete surgical excision is the treatment of choice, with laparoscopy preferred for its minimally invasive nature. More extensive surgical approaches may be necessary in cases of very large, aggressive, or recurrent cysts. Additionally, hormonal therapies—such as tamoxifen, or gonadotropin‐releasing hormone agonists—have been explored in select cases, particularly in women, due to the potential estrogen sensitivity of these cysts, though evidence remains limited [5, 16, 17]. Ultimately, individualized management and multidisciplinary evaluation are essential, given the diagnostic complexity and variable clinical course of PICs.

## 4. Conclusion

PICs are rare, particularly in males without prior abdominal surgery, trauma, or underlying disease. This case highlights an unusual presentation of PIC in a young male, initially misdiagnosed as an adrenal cyst due to its radiological appearance. Such cases underscore the importance of including PICs in the differential diagnosis of cystic abdominal lesions, even in atypical patient profiles. Accurate diagnosis requires a combination of clinical correlation, imaging, and histopathological confirmation to guide appropriate management and avoid unnecessary interventions.

## Consent

Signed informed consent was obtained from the patient for reporting his de‐identified clinical and imaging data.

## Disclosure

All authors reviewed and approved the final version of the manuscript.

## Conflicts of Interest

The authors declare no conflicts of interest.

## Author Contributions

Faezeh Sehatpour, Nekoo Panahi, Mahnaz Pejman Sani, and Maryam Panahi were responsible for drafting the manuscript. Nekoo Panahi and Mahnaz Pejman Sani collaborated closely with Faezeh Sehatpour in managing the patient’s care. The surgical procedure was performed by Shirzad Nasiri. Radiologic evaluations were conducted by Amir Reza Radmard, and the pathological analysis and reporting were done by Farid Kosari.

## Funding

The authors received no specific funding for this work.

## Data Availability

The data that support the findings of this study are available on request from the corresponding author. The data are not publicly available due to privacy or ethical restrictions.
